# THE ROLE OF THE LTSS SCORECARD IN PROMOTING STATE ACCOUNTABILITY FOR CAREGIVER POLICY

**DOI:** 10.1093/geroni/igad104.1642

**Published:** 2023-12-21

**Authors:** Susan Reinhard

**Affiliations:** AARP, Washington, District of Columbia, United States

## Abstract

In 2011, the AARP produced its first LTSS Scorecard in “Raising Expectations: A State Scorecard on Long-Term Services and Supports for Older Adults, People with Physical Disabilities, and Family Caregivers”. This presentation discusses the history, background, and goals of the Scorecard, which examines four key dimensions of state LTSS system performance: affordability and access; choice of setting and provider; quality of life and quality of care; and support for family caregivers. This presentation focuses on the extent to which support for family caregivers was a focus of that first report and how that focus has changed over time. In addition, the presentation will consider the extent to which the Scorecard has proved an effective policy tool in holding states accountable for progress in supporting family caregivers. Has the Scorecard achieved its stated goal to “to spark conversations, galvanize broad-based coalitions, and focus stakeholders’ attention on the factors that most directly impact consumers and their families”? What has AARP learned through its experience of developing and administering the Scorecard, and what changes are being considered with respect to the dimensions pertaining to family caregivers? The presentation will conclude by noting synergies with the work of the RAISE Family Caregiver Advisory Council and the priorities indicated in its National Strategy to Support Family Caregivers.

